# A 24-week multi-component exercise program improves cognition and body composition in older adults with mild cognitive impairment: a randomized controlled trial

**DOI:** 10.3389/fnagi.2025.1711554

**Published:** 2026-01-12

**Authors:** Danming Xu, Xiaochu Wu, Junming Dai, Qing Li, Zhi Wang, Yimin Huang, Yu Zhang, Junjie Cao, Bingxue Li, Yirong Dong, Yanhao Tu

**Affiliations:** 1 Strength and Conditioning Center, Chengdu Sport University, Chengdu, Sichuan, China; 2 National Clinical Research Center for Geriatrics, West China Hospital of Sichuan University, Chengdu, Sichuan, China; 3 School of Physical Education, Chengdu Sport University, Chengdu, Sichuan, China

**Keywords:** body composition, cognitive function, multicomponent exercise intervention, randomized controlled trial (RCT), skeletal muscle mass

## Abstract

**Background:**

This study investigated whether a 24-week, community-based multicomponent exercise intervention (MCEI) can improve body composition and cognitive function in older adults with mild cognitive impairment (MCI).

**Methods:**

In this single-center, parallel-group randomized controlled trial (RCT), 64 community-dwelling adults aged 65–75 years with MCI characterized by Mini-Mental State Examination (MMSE) ≥ 24, Montreal Cognitive Assessment (MoCA) ≤ 26, Clinical Dementia Rating (CDR) = 0.5, low skeletal muscle mass were randomly allocated (1:1) to a MCEI (aerobic, resistance and balance training, 3 × 60 min/week) or to a usual-activity control (UAC) group receiving weekly health education. Primary outcomes were skeletal muscle mass (SMM), fat-mass index (FMI), MMSE and MoCA; secondary outcomes included skeletal muscle index (SMI), basal metabolic rate (BMR), Instrumental Activities of Daily Living (IADL) and Animal Fluency Test (AFT). Assessments were conducted at baseline and within 1 week post-intervention by trained, blinded assessors. Intervention effects were examined with a 2 (group) × 2 (time) repeated-measures analysis of variance (ANOVA), reporting partial eta-squared (η^2^) as the effect-size estimate.

**Results:**

A significant Group × Time interaction was observed for SMM (*p* < 0.001, η^2^ = 0.195) and FMI (*p* = 0.003, η^2^ = 0.153), indicating differential changes between groups; with significant improvements observed only in the MCEI group. SMI showed no significant interaction effect (*p* = 0.270, η^2^ = 0.021), whereas no significant interactions were found for MMSE, MoCA, or AFT (*p* ≥ 0.18, η^2^ ≤ 0.03).

**Conclusion:**

A 24-week community-based multicomponent exercise program safely increased skeletal muscle mass and reduced fat mass in older adults with MCI, but did not produce measurable improvements on screening-level cognitive measures. Future studies with longer duration, larger samples, and inclusion of cognitive challenges are warranted to clarify exercise–cognition interactions and establish dose–response relationships for both body composition and domain-specific cognition.

**Clinical trial registration:**

Identifier ChiCTR2000035012.

## Introduction

China is among the fastest-aging countries in the world, with the prevalence of mild cognitive impairment (MCI) in adults aged 65 years and above reaching approximately 19% (Shi et al., 2022). Aging itself represents the strongest non-modifiable risk factor for late-life cognitive decline and neurodegenerative diseases. As an intermediate stage between normal aging and dementia, MCI significantly increases the risk of progression to Alzheimer’s disease (Busse et al., 2003; Ravaglia et al., 2008), adversely affects quality of life in older adults (Khan et al., 2023), and imposes substantial economic and caregiving burdens on families and society (Teveles, 2023). In the persistent absence of effective pharmacological interventions capable of halting cognitive decline, identifying safe and scalable strategies to preserve cognitive and brain health in older adults has become an urgent priority (Luo et al., 2021).

Among various intervention strategies, non-pharmacological approaches have emerged as central methods for enhancing cognitive function in older adults. These strategies can be broadly categorized into three groups: (1) physical exercise, (2) cognitive training, and (3) social engagement (Silva et al., 2024; Venegas-Sanabria et al., 2020). Physical exercise has received particular attention due to its multi-target, multi-system benefits. Accumulating evidence from systematic reviews and meta-analyses indicates that regular exercise not only improves cardiometabolic and physical health but also enhances cerebral perfusion, elevates brain-derived neurotrophic factor (BDNF) levels, mitigates chronic neuroinflammation, and subsequently benefits memory, attention, and global cognitive function (Falck et al., 2019; Zhou et al., 2022; Mielniczek and Aune, 2024; Titus et al., 2021; Ruiz-González et al., 2021). In contrast, cognitive training primarily yields domain-specific gains (Chan et al., 2024; Cabreira et al., 2024), whereas social engagement largely contributes to emotional and psychosocial well-being (Li et al., 2024). Collectively, physical exercise is considered one of the most cost-effective non-pharmacological strategies to promote healthy aging.

Recently, multicomponent exercise intervention (MCEI) and cognitive–physical combined training have gained increasing attention (Rieker et al., 2022; Meng et al., 2021). These programs typically integrate aerobic, resistance, and balance exercises with varying levels of cognitive challenge. Previous studies have suggested that such comprehensive interventions may confer broad cognitive benefits in older adults, including older adults with MCI, potentially improving attention, memory, executive functions (EFs), and daily functional abilities (Li et al., 2025; Lai et al., 2025; Chen et al., 2023). Attention, executive functions, and related cognitive domains are among the areas most likely to benefit, although the extent of improvement can vary across studies.

Despite promising findings, several limitations remain in the current literature. First, many intervention trials are relatively short (typically ≤12 weeks), limiting the ability to assess the long-term effects of exercise on cognition and body composition. Second, while body composition indicators—such as skeletal muscle mass and fat mass—serve as important markers of physical and metabolic health in older adults, most studies focus predominantly on cognitive outcomes, often overlooking body composition despite its relevance to cognitive aging. Third, long-term, community-based randomized controlled trials (RCTs) that simultaneously assess cognitive function and body composition in older adults remain scarce.

To address these gaps, the present study implemented a 24-week community-based MCEI randomized controlled trial (RCT) to examine whether a multicomponent exercise intervention could simultaneously improve cognitive function and body composition in older adults with MCI. We hypothesized that participants in the intervention group would show greater improvements than those in a usual-activity control group.

## Methods

### Study design and ethics

This single-center, community-based RCT was conducted in accordance with the SPIRIT 2013 statement and reported following the CONSORT 2025 guidelines. The trial was led by West China Hospital, Sichuan University, and implemented in two urban communities in Chengdu. A parallel-group design was used, in which eligible participants were randomly allocated (1:1) to either the MCEI group or the usual activity control (UAC) group. The 24-week intervention consisted of three 60 min supervised MCEI sessions per week, while the UAC group maintained habitual activities and attended weekly health education sessions. No interim assessments were conducted; a comprehensive endpoint evaluation was performed within 1 week after the intervention (December 1–7, 2023) by trained, blinded assessors. Participants remained unaware of group allocation. To ensure consistency, all exercise and assessment sessions were scheduled at the same time of day (08:00–10:00). Participants were instructed to avoid caffeine, alcohol, and strenuous physical activity for 24 h prior to assessments, consume a standardized light meal 2 h beforehand, and maintain hydration (≥1,500 mL/day), with fluid intake recorded prior to each assessment.

### Participants

### Sample size

An *a priori* power analysis (G*Power 3.1, repeated-measures mixed ANOVA, within–between interaction) indicated that a medium effect size of *f* = 0.25, *α* = 0.05, and power = 0.80 required a minimum of 34 participants. We enrolled 64 participants (32 per group), yielding an actual power >0.80.

### Recruitment and screening

A four-stage recruitment protocol was implemented to maximize 24-week retention by addressing common causes of early dropout and enhancing participants’ engagement.

(1) Community outreach: Local health stations and senior-citizen centers collaborated to establish preliminary trust, which reduced immediate post-enrolment withdrawal.
(2) Spaced screening: Assessment sessions were scheduled 2–5 days apart to avoid cognitive overload and early dropout due to fatigue.
(3) Individualized feedback: Certified geriatricians provided face-to-face feedback to enhance participants’ perceived safety and the value of participation, thereby strengthening commitment.
(4) Proactive exclusion of high-risk individuals: The principal investigator integrated medical, logistical, and family-support information to identify and exclude participants with high attrition risk (e.g., transportation or health constraints), keeping expected loss below 5%.

Together, these steps ensured that participants felt safe, supported, and capable of completing the full 24-week intervention.

### Inclusion and exclusion criteria

Eligibility was based on the 2018 Chinese Guidelines for the Diagnosis and Treatment of Dementia and Cognitive Impairment (Part V: MCI) and other standardized frameworks (Petersen et al., 2018.).

#### Inclusion criteria

(1) Aged 65–75 years, community-dwelling, with self-reported or clinically confirmed memory complaints.
(2) Clinical Dementia Rating (CDR) = 0.5, Global Deterioration Scale (GDS) 2–3, Mini-Mental State Examination (MMSE) ≥ 24, Montreal Cognitive Assessment (MoCA) ≤ 26, Animal Fluency Test (AFT) ≥ 15, Instrumental Activities of Daily Living (IADL) ≥ 6, preserved basic activities of daily living.
(3) Skeletal Muscle Index (SMI) < 7.0 kg·m^−2^ (men) or < 5.7 kg·m^−2^ (women), Skeletal Muscle Mass (SMM) < 26.3 kg (men) or < 18.9 kg (women), Fat Mass Index (FMI) < 7.0 kg·m^−2^ (men) or < 14.0 kg·m^−2^ (women).
(4) Able to ambulate independently, with moderate-intensity exercise tolerance confirmed by cardiopulmonary exercise testing and Physical Activity Readiness Questionnaire (PAR-Q).
(5) Signed informed consent.

#### Exclusion criteria

Irreversible visual impairment, severe cardio-cerebrovascular or uncontrolled metabolic diseases, participation in structured multicomponent exercise within the past 3 months, or clinical diagnosis of dementia.

### Randomization and allocation

A two-stage randomization procedure was employed (Wu et al., 2023):

Community-level: Two urban communities served as recruitment sites only and were not used as randomization units.
Individual level: Eligible participants from both communities were randomized (1:1) to the MCEI or UAC group using computer-generated variable block sizes (4 and 6), stratified by sex.

The sequence was generated by an independent statistician and stored in a password-protected file. Allocation concealment was maintained until baseline assessments were completed. Participants were not blinded, but outcome assessors and data analysts were.

### Ethics approval and consent

All procedures were conducted in accordance with the Declaration of Helsinki and approved by the Medical Ethics Committee of West China Hospital, Sichuan University (approval No. 2020-287). The trial was registered with the Chinese Clinical Trial Registry (ChiCTR2000035012).

### Intervention protocol

#### Multicomponent exercise intervention

A fixed 60 min template was used for all 72 supervised sessions (Mon/Wed/Fri, 08:30–09:30). Table 1 presents a minute-by-minute blueprint for Phase A (Week 3, Monday); identical structure was applied in Phases B and C with progressed loads (see Appendix 1). The minute-by-minute blueprint for Phase A (Week-3, Monday); identical structure was applied in Phases B & C with progressed loads detailed in Appendix 1.

**Table 1:** Example 60-minute session blueprint (Phase A, Week-3, Monday).

Time (min)	Sequence & load	HR/RPE	Rest	Safety notes
00–02	BP & HR screen	–	–	Abnormal→seated track
02–10	Warm-up	50% HRmax	30 s	Non-slip floor; 2 m spacing
10–25	Block A-Baduanjin	55% HRmax	30 s water	Tablet flashes if >80 %HRmax
25–37	Block B-Resistance	RPE 11–12	45 s intra	Auto “half-set” after3fails
37–45	Block C-Balance	≤60% HRmax	30 s walk	Fall: stop-ice-report ≤24 h
45–53	Cool-down	≤50% HRmax	–	Photo taken; missing
53–60	Hydration, RPE	–	–	Upload within 24 h

Target HR zones 50–75% HRmax; intra-set rest 45 s, between-set 60s; real-time HR monitored at 1 Hz.

#### Intensity progression (see Appendix 1 for full tables)

Load increased every 4 weeks: +1 kg dumbbell or medicine ball, upper HR ceiling 75% HRmax. Three consecutive full-set failures → automatic “half-set”; two consecutive “half-set” sessions triggered individual reassessment.

#### Time feasibility

Pilot runs (12 older adults × 3 sessions) showed the main exercise block was completed in 46.8 ± 1.2 min; total 8 min warm-up + 47 min main + 5 min cool-down fits exactly 60 min without compression.

#### Safety measures beyond ACSM guidelines

(1) Pre-session health checklist and accident insurance coverage.
(2) AED and first-aid kit on site; instructors certified in first aid.
(3) Real-time HR alert at 80% HRmax; automatic load reduction or extra rest.
(4) Post-session “three-in-one” e-package (QR sign-in, HR.csv, de-identified photo); missing components supplemented within 24 h.

#### Fidelity and adherence audit

20% of sessions were randomly audited using a 19-item checklist (*α* = 0.88); mean score 94.3% (SD 3.1). Adherence was defined as ≥58/72 sessions (80%) meeting “on-target” criteria (≥20 min within prescribed HR zone + full 60 min attendance). Weekly SMS feedback was sent to participants.

### Usual activity control group

Participants in the UAC group continued their regular daily activities and attended weekly 1 h health education sessions for 24 weeks. Educational content was prepared by the project team, with printed handouts distributed. During the pilot phase, experts in medicine and exercise science delivered in-person guidance, ensuring engagement without structured physical training.

### Outcome assessments

All assessments were conducted at baseline and within 1 week post-intervention under standardized conditions by trained, blinded assessors.

Cognitive testing was performed individually in a quiet room by two certified neuropsychologists:

(1) MMSE: Assesses global cognition, including orientation, registration, attention, calculation, recall, language, and visuospatial skills.
(2) MoCA: Evaluates multiple cognitive domains, including EFs, attention, abstraction, and visuospatial abilities through tasks such as trail-making, cube and clock drawing. A score ≤26 indicates MCI.
(3) AFT: Measures semantic memory and verbal processing speed by counting the number of animals named within 90 s.
(4) IADL: Assesses functional ability across seven complex daily tasks that require executive function and memory application.

Body composition was assessed with the InBody 770 multi-frequency BIA after ≥ 8 h fasting, emptied bladder, removal of metal, full limb-electrode contact, daily calibration (impedance error <1%), room temperature 23 ± 1 C.

### Heart rate monitoring

Heart rate (HR) was continuously monitored during each MCEI session using Huawei Band 6 (1 Hz sampling). Data were exported to determine time spent in target HR zones.

### Outcome measures

#### Primary outcome

Primary outcomes included MMSE, MoCA, SMM, and FMI. Interrelationships among these indicators were also explored.

#### Secondary outcomes

Secondary outcomes comprised AFT, IADL, SMI, and BMR. Interrelationships among these variables were similarly examined.

### Statistical analysis

**Figure 1:**
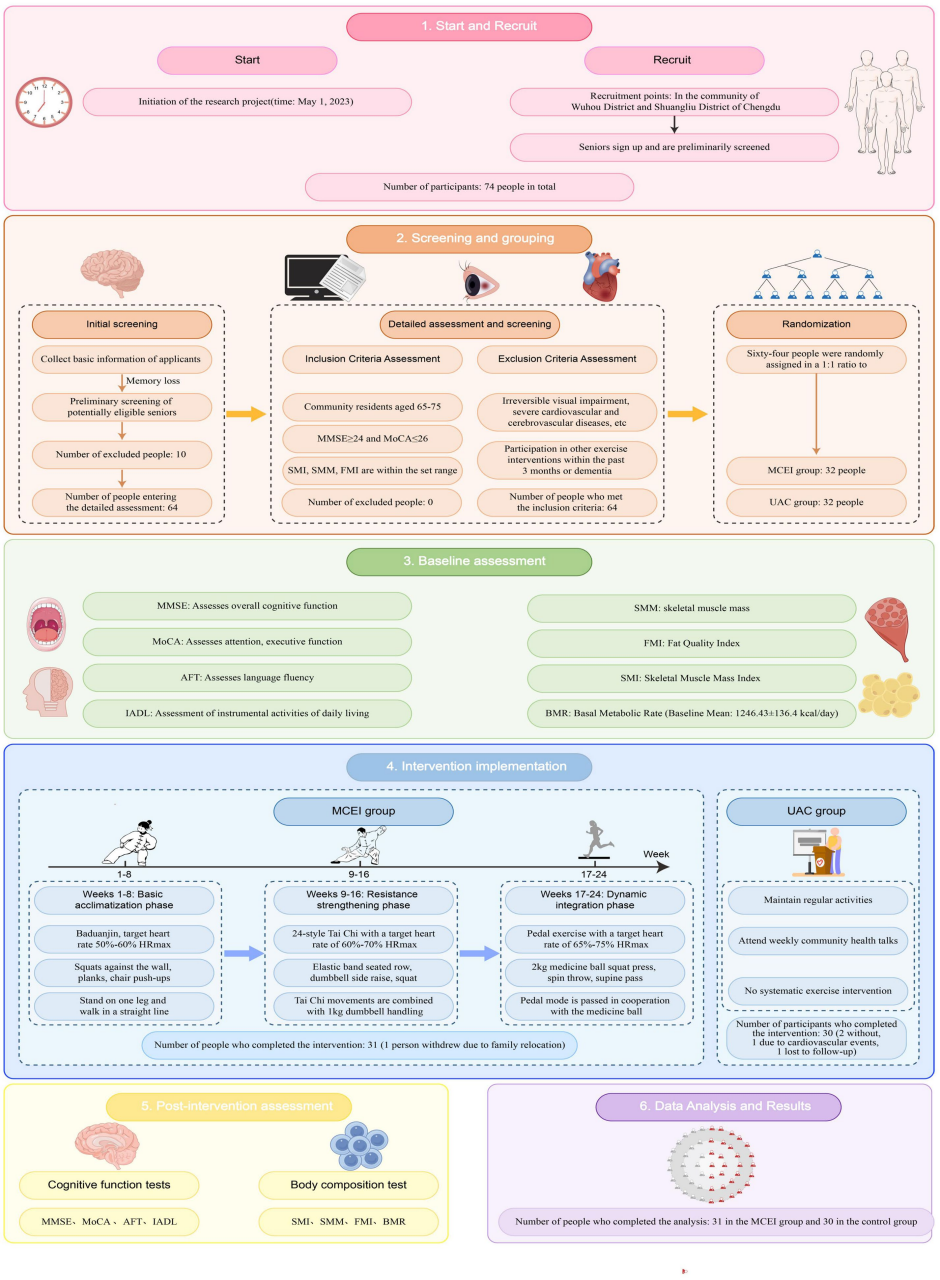
Study flowchart. Flow of participants through the study, including recruitment, screening, randomization, intervention, and assessment phases. MCEI, multi-component exercise intervention; UAC, usual activity control.

Statistical analyses were performed using SPSS version 26.0 (IBM Corp., Armonk, NY, United States). An intention-to-treat (ITT) approach was adopted, retaining participants with at least one post-baseline assessment and imputing missing values using the last-observation-carried-forward (LOCF) method. A per-protocol (PP) sensitivity analysis (attendance ≥ 80%) was also conducted to verify robustness. Outliers were identified using the box-plot method (1.5 × IQR) and Winsorized to the nearest non-outlier value. Normality was assessed via Shapiro–Wilk tests, and homogeneity of variances using Levene’s test. Baseline characteristics were compared between groups using independent-samples *t*-tests (for normally distributed data) or Mann–Whitney U tests (for non-normal data), while categorical variables were analyzed using χ^2^ tests. For primary and secondary outcomes, a 2 (Group) × 2 (Time) repeated-measures ANOVA was conducted using Type-III sums of squares. The assumption of sphericity was automatically met given the two measurement points. Partial eta-squared (η^2^) was reported for main and interaction effects, and Cohen’s d (calculated using pooled baseline SD) was provided for change scores. Following significant interactions, post-hoc pairwise comparisons were performed with a Bonferroni correction. All tests were two-tailed, with statistical significance set at *p* < 0.05 (Figure 1).

## Result

### CONSORT diagram

**Figure 2:**
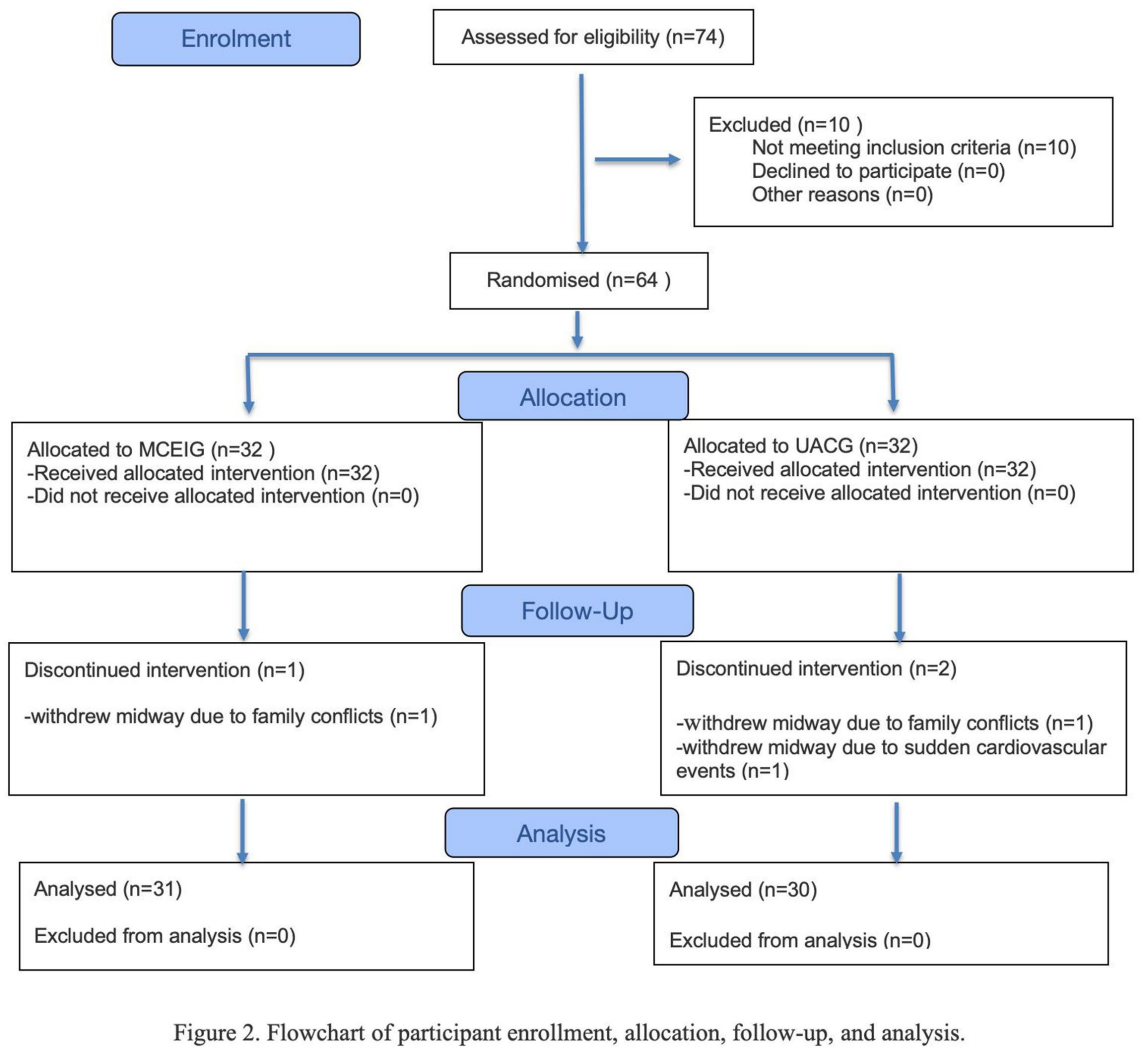
Flowchart of participant enrollment, allocation, follow-up, and analysis.

Figure 2 shows that 95.3% of participants (61/64) completed the endpoint assessment; three withdrawals occurred for family conflicts (*n* = 2) or acute cardiovascular events (*n* = 1).

### Baseline characteristics of the study population

Table 2 summarizes baseline demographics and key variables. No significant between-group differences were observed in age, sex distribution, height, weight, BMI, MMSE, MoCA, AFT, IADL, SMI, SMM, FMI, or BMR (all *p* > 0.05), indicating successful randomization.

**Table 2:** Baseline characteristics of participants.

Variable	All participants (*n* = 61)	MCEIG (*n* = 31)	UACG (*n* = 30)	*p*-value
Demographics				
Mean (SD) age (years)	68.49 (4.4)	68.63 (5.31)	69.37 (4.44)	0.540
Gender (%)				
Male	19 (31.1)	9 (29.00)	10 (33.30)	0.716
Female	41 (68.9)	22 (71.00)	20 (66.70)	
Mean (SD) Height (cm)				
Male	162.90 (4.8)	162.26 (5.91)	163.56 (4.04)	0.620
Female	152.30 (4.4)	153.07 (3.72)	151.50 (5.15)	0.289
Mean (SD) Weight (Kg)				
Male	63.65 (7.74)	61.98 (7.89)	64.50 (7.86)	0.570
Female	55.45 (6.86)	56.51 (7.86)	54.63 (6.02)	0.391
Mean (SD) BMI (kg/m)				
Male	23.98 (2.81)	23.51 (2.30)	24.4 (3.27)	0.639
Female	23.87 (2.40)	23.96 (2.71)	23.77 (2.06)	0.645
Mean (SD) cognitive function indicators				
MMSE Score (/30)	26.85 (2.10)	26.90 (2.50)	26.80 (1.63)	0.913
MoCA score (/30)	20.38 (3.48)	20.77 (3.64)	20.07 (3.38)	0.504
AFT score	26.26 (5.25)	27.03 (6.15)	25.47 (4.08)	0.28
IADL (/8)	7.54 (0.99)	7.52 (0.96)	7.57 (1.04)	0.783
Mean (SD) body composition indicators				
SMI (kg·m⁻^2^)	5.83 (0.60)	5.84 (0.64)	5.82 (0.58)	0.759
SMM (kg)	19.32 (2.82)	19.20 (2.88)	19.44 (2.82)	0.879
FMI (kg·m⁻^2^)	9.05 (1.62)	8.89 (1.96)	9.13 (1.21)	0.591
BMR (kcal·day⁻^1^)	1246.43 (136.4)	1266.26 (140.25)	1225.93 (131.67)	0.261

Data are presented as number (percentage) of participants unless otherwise specified. MMSE, Mini-Mental State Examination; MoCA, Montreal Cognitive Assessment; IADL, Instrumental Activities of Daily Living; AFT, Animal Fluency Test; SMI, Skeletal Muscle Mass Index; SMM, Skeletal Muscle Mass; FMI, Fat Mass Index; BMR, Basal Metabolic Rate; SD, Standard Deviation; *p* value, the result of statistical hypothesis testing, indicating whether the observed differences are statistically significant.

### Impact of the 24-week multi-component exercise program on primary outcomes

The results revealed a statistically significant Group × Time interaction effect for SMM (*F*(1, 59) = 12.08, *p* ≤ 0.001, η^2^ = 0.166). Simple effects analysis indicated that the exercise group exhibited a significant pre-to-post difference in SMM (*F*(1, 59) = 14.32, *p* ≤ 0.001, η^2^ = 0.195), whereas no significant change was observed in the control group (*F*(1, 59) = 0.21, *p* = 0.65, η^2^ = 0.004). Furthermore, post-intervention SMM in the exercise group was significantly higher than that in the control group (*F*(1, 59) = 5.92, *p* = 0.018, η^2^ = 0.091).

Similarly, for FMI, a significant Group × Time interaction was found (*F*(1, 59) = 9.89, *p* = 0.003, η^2^ = 0.140). Simple effects analysis showed a significant pre -to-post difference in FMI in the exercise group (*F*(1, 59) = 10.67, *p* = 0.002, η^2^ = 0.153), but not in the control group (*F*(1, 59) = 0.15, *p* = 0.70, η^2^ = 0.003). Post-intervention FMI was also significantly lower in the exercise group compared to the control group (*F*(1, 59) = 4.33, *p* = 0.042, η^2^ = 0.068) (Tables 3–5).

**Table 3:** Impact of the 24-week multi-component exercise program on cognitive and body-composition outcomes at two timepoints (Group × Time) in older adults with MCI (mean ± SD) (*N* = 61).

Overtime measure	Group factor effect		Time factor effect		Group × Time effect		Measure time	
	η2	*P*-value	η2	*P*-value	η2	*P*-value	T0 (Pre)	T1 (Post)
*MMSE (points)*	0.007	0.511	0.001	0.841	0.011	0.418	–	–
MCEIG							26.87 ± 2.54	26.53 ± 2.83
UACG							26.80 ± 1.63 [0.02]	27.17 ± 1.58 [0.174]
*MoCA (points)*	0.012	0.396	0.252	0.000**	0.033	0.161	–	–
MCEIG							20.77 ± 3.64	22.50 ± 3.66
UACG							20.07 ± 3.38 [0.12]	20.60 ± 3.24 [0.32]
*AFT (points)*	0.061	0.054	0.012	0.402	0.002	0.720	–	–
MCEIG							26.93 ± 6.23	28.07 ± 6.46
UACG							25.47 ± 4.07 [0.20]	25.50 ± 3.95 [0.33]
*IADL (points)*	0.006	0.552	0.020	0.281	0.002	0.730	–	–
MCEIG							7.50 ± 0.97	7.70 ± 0.70
UACG							7.57 ± 1.04 [0.05]	7.53 ± 1.04 [0.15]
*SMI (kg·m⁻* ^2^ *)*	0.020	0.283	0.179	0.001**	0.021	0.270	–	–
MCEIG							5.84 ± 0.63	6.02 ± 0.73
UACG							5.82 ± 0.58 [0.06]	5.91 ± 0.54 [0.13]
*SMM (kg)*	0.014	0.369	0.140	0.003*	0.166	0.001**	–	–
MCEIG							19.32 ± 2.85	19.99 ± 2.96
UACG							19.44 ± 2.82 [0.03]	19.39 ± 2.70 [0.14]
*FMI (kg·m⁻* ^2^ *)*	0.001	0.001**	0.087	0.021*	0,140	0.003*	–	
MCEIG							8.99 ± 1.99	8.52 ± 1.70
UACG							8.84 ± 1.59 [0.21]	8.74 ± 1.73 [0.17]
*BMR (kcal·day⁻* ^1^ *)*	0.031	0.175	0.000	0.864	0.019	0.289	–	–
MCEIG							1271.23 ± 139.83	1283.70 ± 149.24
UACG							1225.93 ± 131.67 [0.21]	1222.50 ± 149.13 [0.2]

Data are presented as mean ± standard deviation. Repeated-measures ANOVA was used to examine the Group×Time interaction, with Group (Intervention vs. Control) as the between-subjects factor and Time (Pre vs. Post) as the within-subjects factor. Ges, generalized eta-squared; *P* < 0.05 was considered statistically significant; MMSE, Mini-Mental State Examination; Moca, Montreal Cognitive Assessment; AFT, Animal Fluency Test; IADL, Instrumental Activities of Daily Living; SMI, Skeletal Muscle Index; SMM, Skeletal Muscle Mass; FMI, Fat Mass Index; BMR, Basal Metabolic Rate; T0, baseline; T1, post-intervention.

**Table 4:** Simple-effect analysis, between-group difference (mean ± SD) (*N* = 61).

Outcome measures	Between-group comparison	T0			T1		
		Difference (95% CI)	*t*	*P* value	Difference (95%CI)	*t*	*P*-value
SMI (kg·m⁻^2^)	MCEIG:UACG	0.02 (−0.28, 0.32)	0.15	0.882	0.09 (0.04, 0.14)	3.71	<0.001**
SMM (kg)	MCEIG:UACG	−0.24 (−1.45, 0.97)	−0.40	0.694	0.46 (0.05, 0.87)	2.21	0.032*
FMI (kg·m⁻^2^)	MCEIG:UACG	−0.15 (−0.93, 0.63)	−0.38	0.707	−0.47 (−1.05, 0.11)	−1.59	0.118

*Indicates significant intra−/inter group differences before/after intervention (*p* < 0.05);**indicates extremely significant differences (*p* < 0.01); Cohen’s d (95% CI) = effect size with 95% confidence interval. MMSE, Mini-Mental State Examination; Moca, Montreal Cognitive Assessment; AFT, Animal Fluency Test; IADL, Instrumental Activities of Daily Living; SMI, Skeletal Muscle Index; SMM, Skeletal Muscle Mass; FMI, Fat Mass Index; BMR, Basal Metabolic Rate; T0, baseline; T1, post-intervention.

**Table 5:** Simple-effect analysis, within-group change (mean ± SD) (*N* = 61).

Outcome measures	MCEIG			UACG		
	*T*	*P*	Difference 95% CI	*T*	*P*	Difference 95% CI
SMI (kg·m⁻^2^)						
T0:T1	−2.961	0.006**	0.53 (−0.28, −0.05)	−2.050	0.049*	0.37 (−0.17, −0.00)
SMM (kg)						
T0:T1	−3.521	0.001**	0.63 (−1.02, −0.27)	0.799	0.431	0.15 (−0.09, 0.20)
FMI (kg·m⁻^2^)						
T0:T1	3.364	0.002*	0.60 (0.18, 0.76)	0.386	0.702	0.08 (−0.32, 0.48)

*Indicates significant intra−/inter group differences before/after intervention (*p* < 0.05); ** indicates extremely significant differences (*p* < 0.01); Cohen’s d (95% CI) = effect size with 95% confidence interval. SMI, Skeletal Muscle Index; SMM, Skeletal Muscle Mass; FMI, Fat Mass Index; BMR, Basal Metabolic Rate; T0, baseline; T1, post-intervention.

### Impact of the 24-week multi-component exercise program on secondary outcomes

For SMI, no significant Group × Time interaction was observed (*F*(1, 59) = 1.21, *p* = 0.270, η^2^ = 0.021), whereas a significant main effect of Time was found (*F*(1, 59) = 12.86, *p* = 0.001, η^2^ = 0.179). Simple-effects analysis showed a significant pre-to-post increase in the exercise group (*F*(1, 59) = 8.77, *p* = 0.006) and a smaller but significant increase in the control group (*F*(1, 59) = 4.20, *p* = 0.049). Between-group comparisons indicated no baseline difference (*F*(1, 59) = 0.02, *p* = 0.882) but a significant post-intervention difference favoring exercise (*F*(1, 59) = 13.76, *p* < 0.001).

## Discussion

The 24-week multicomponent exercise program significantly increased SMM and decreased FMI in community-dwelling older adults with MCI. No Group × Time interaction was observed for MMSE or MoCA total scores; both are screening-level instruments with limited sensitivity to detect subtle cognitive changes (D’Ignazio et al., 2025; Roalf et al., 2012). The negligible between-group difference on the AFT suggests that observed changes were task-specific rather than indicative of broader cognitive improvement. Within-group improvements in MoCA scores should be interpreted cautiously, as they may reflect practice effects or random variation.

Exercise is hypothesized to support cognitive reserve through enhanced neuroplasticity, increased cerebral blood flow, up-regulation of BDNF, and reduced chronic inflammation (Marmeleira, 2013; Cabral et al., 2019; Wang et al., 2025). However, in the present trial, no significant cognitive effects were detected, which aligns with prior studies reporting ceiling effects that can obscure small changes (Baker et al., 2010; Suzuki et al., 2012).

Body-composition changes were intervention-specific. Skeletal muscle acts as an endocrine organ, releasing myokines that may modulate neuroinflammation (Isaac et al., 2011), whereas excess adipose tissue is associated with pro-inflammatory states linked to brain atrophy (Walston, 2012). The present 72-session protocol produced a “muscle-gain, fat-loss” profile exceeding typical fixed-intensity aerobic benchmarks (Djosic et al., 2025) and meeting inflammatory thresholds proposed for MCI cohorts (Cadore et al., 2013; Zhang and Wang, 2021). Minimal changes in the control group indicate that habitual physical activity alone is insufficient to alter SMM or FMI over 24 weeks (Lim et al., 2023), providing dose-reference values for future trials.

This study had several limitations. The absence of a cognitive-only arm precludes quantifying the independent contribution of cognitive stimulation. The over-representation of women may have influenced results through sex-specific social baselines (Wheatley et al., 2021; Rai et al., 2020). The protocol did not include dual-task or cognitive-enrichment components, which may be necessary to elicit broader transfer effects, including potential improvements in executive functions. Future trials could incorporate dual-task challenges or executive function-specific tasks (e.g., Stroop, N-back) to better capture cognitive outcomes beyond screening tools. Additionally, the lack of blood-based biomarkers such as BDNF limits our ability to assess potential neurobiological mechanisms underlying the observed effects, suggesting that future studies should incorporate biomarker measurements to strengthen mechanistic interpretations.

## Conclusion

In summary, the 24-week community-based multicomponent exercise program was safe, well-tolerated, and effective in increasing SMM and reducing FMI in older adults with MCI. No significant improvements were observed on screening-level cognitive outcomes (MMSE/MoCA), and AFT changes were task-specific. Future studies should consider three-arm designs (exercise-only, cognitive-only, combined) with dual-task or cognitive-enrichment components to explore exercise-cognition synergies and establish dose–response relationships for both body composition and domain-specific cognitive outcomes. The present 60 min, thrice-weekly, ≥24-week template can be readily implemented in community medical-fitness services, offering a low-cost, scalable approach for managing SMM and FMI in older adults with MCI.

## Data Availability

The trial protocol and statistical analysis plan presented in this study are available through the following BMC Neurology citation: https://doi.org/10.1186/s12883-023-03390-5.

## Glossary

AD: Alzheimer’s disease
AFT: Animal Fluency Test
aMCI: amnestic mild cognitive impairment
BDNF: brain-derived neurotrophic factor
BMR: basal metabolic rate
CDR: Clinical Dementia Rating
CONSORT: Consolidated Standards of Reporting Trials
FMI: fat-mass index
GDS-15: Geriatric Depression Scale-15
HRmax: maximum heart rate
IADL: Instrumental Activities of Daily Living
MCEI: multicomponent exercise intervention
MCI: mild cognitive impairment
MMSE: Mini-Mental State Examination
MoCA: Montreal Cognitive Assessment
PAR-Q: Physical Activity Readiness Questionnaire
PI: principal investigator
RCT: randomized controlled trial
RM: repetition maximum
RPE: rating of perceived exertion
SMI: skeletal muscle index
SMM: skeletal muscle mass
STAI: State–Trait Anxiety Inventory
UAC: usual activity control
