# Supplementary figures and images for: A 24-week multi-component exercise program improves cognition and body composition in older adults with mild cognitive impairment: a randomized controlled trial
Source: Front Aging Neurosci. 2026 Jan 12;17:1711554. doi: 10.3389/fnagi.2025.1711554 (PMC12832871; doi:10.3389/fnagi.2025.1711554)

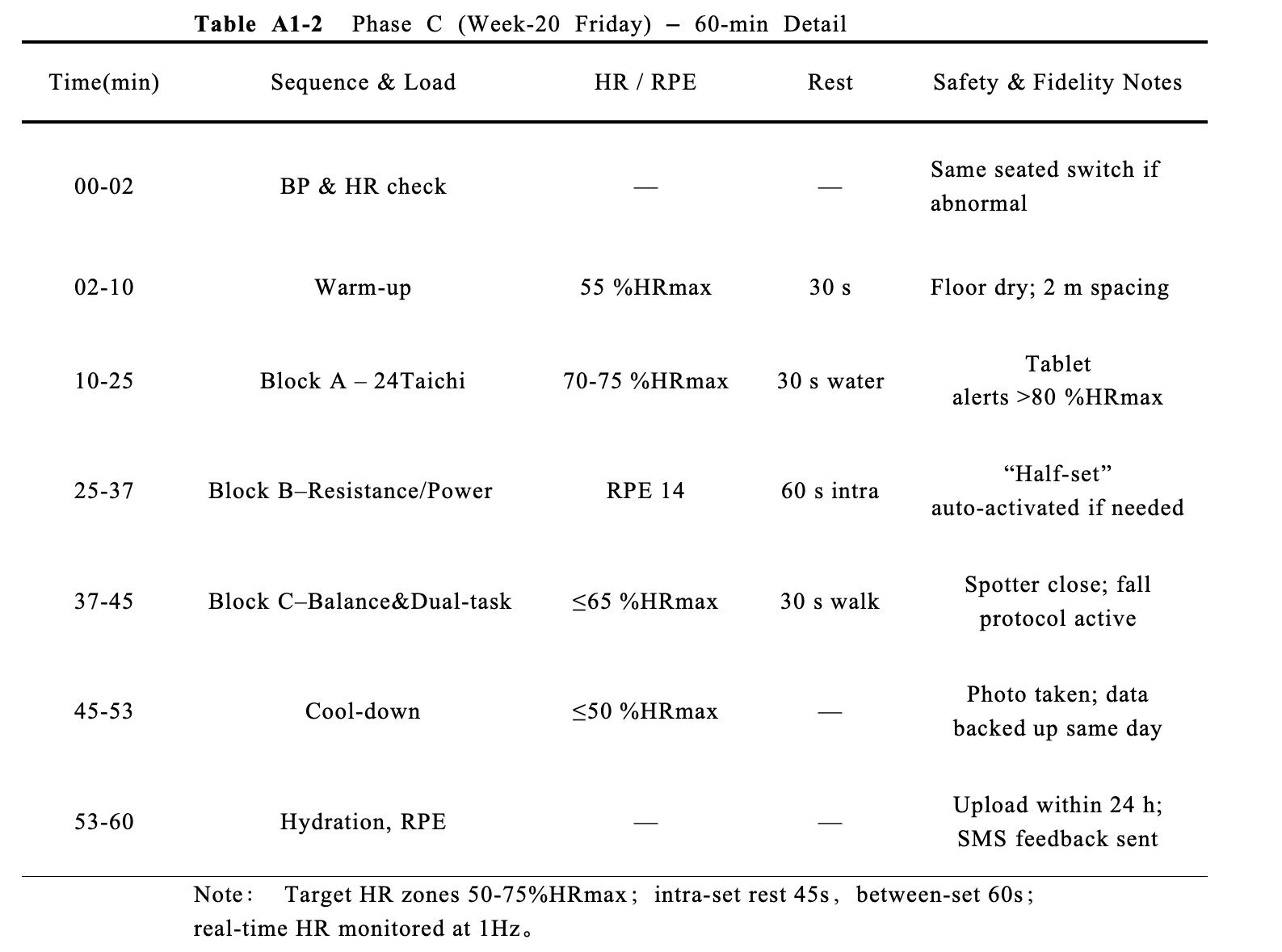

Supplement: Supplementary file 1 [file Data_Sheet_1.zip › Appendix 1/Table A1-2_01.jpg]

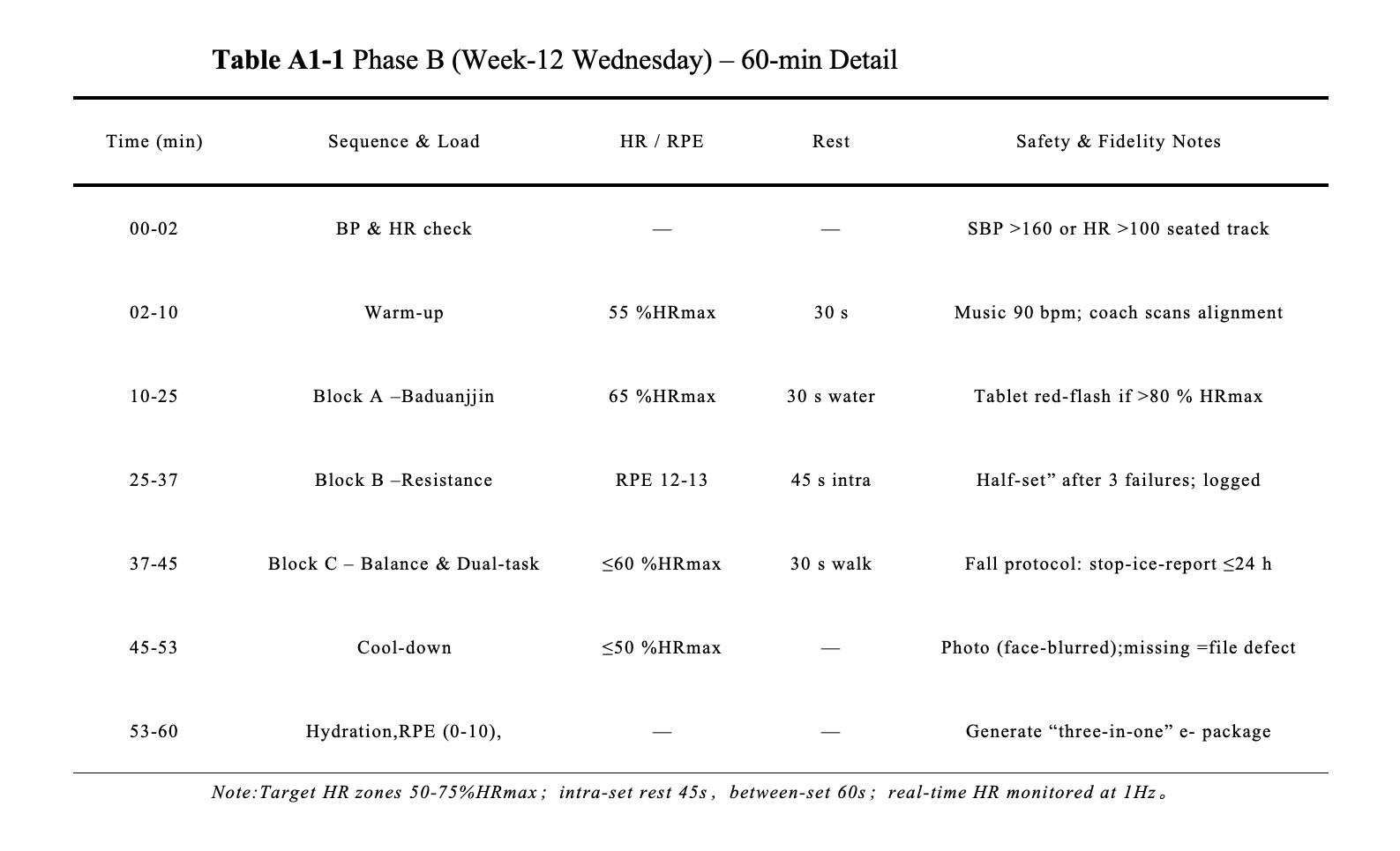

Supplement: Supplementary file 1 [file Data_Sheet_1.zip › Appendix 1/Table A1-1_01.jpg]
